# A FOS/NFKB1-associated Hofbauer cell subset mediates placental niche dysregulation in early-onset fetal growth restriction

**DOI:** 10.3389/fcell.2026.1827600

**Published:** 2026-05-29

**Authors:** Yuanjie Sun, Haosheng Lin, Wenqian Zhu, Liuchang Niu, Yilin Li, Yuan Huang, Qingxian Chang

**Affiliations:** Department of Obstetrics and Gynecology, Nanfang Hospital, Southern Medical University, Guangzhou, Guangdong Province, China

**Keywords:** early-onset FGR, fos, hofbauer cells, NFKB1, snRNA-seq, trophoblast invasion

## Abstract

**Background/Objective:**

Early-onset fetal growth restriction (FGR) is a severe pregnancy complication caused by placental dysfunction. Although superficial trophoblast invasion and sterile inflammation are recognized as characteristics, the specific cellular driving factors and molecular mechanisms that lead to the dysregulation of the immune-trophoblast microenvironment remain unclear.

**Methods:**

We constructed a high-resolution single-nucleus transcriptomic (snRNA-seq) atlas of placental tissues from patients with early-onset FGR and their matched normal controls. By integrating bioinformatics methods, such as pseudotime trajectory inference, gene regulatory network analysis (SCENIC), and intercellular communication modeling (CellChat), along with *in vitro* validation approaches, such as hypoxia-induced macrophage models, recombinant protein stimulation, and RT-qPCR, we identified specific changes associated with this disease.

**Results:**

Our research revealed impaired differentiation trajectories of trophoblast cells, characterized by the absence of a critical intermediate state essential for acquiring invasiveness. At the same time, we identified a pathogenic Hofbauer cell subset (HBC5), which increases in number in FGR and exhibits high regulon activity of FOS and NFKB1. *In vitro* experiments have confirmed that hypoxia triggers the polarization of HBC5-like macrophages, leading to the upregulation of pro-inflammatory factors such as CCL4. Furthermore, the CellChat analysis combined with functional validation indicates that the CCL4 produced by HBC5 significantly induces mitochondrial stress markers *GDF15* and *APP* in trophoblast cells, resulting in dysregulation of the placental microenvironment. Mechanistically, unlike physiological HBC that provide trophic support, HBC5 shows impaired secretion of IGF1, and implements dual blockade of inflammation and metabolism on Extravillous Trophoblasts (EVT) and the vascular system through CCL chemokines and NAMPT signaling pathways. Moreover, we have elucidated a vicious cycle: trophoblast cells under stress conditions release danger signals such as GDF15 and APP, which may continuously maintain the pathogenic immune polarization state of HBC5, and polarized HBC5 in turn stimulates trophoblast cells.

**Conclusion:**

Our study reveals that the HBC5 subset with high FOS/NFKB1 regulon activity acts as a key mediator of placental niche dysregulation. The identified HBC5-CCL4-Trophoblast stress axis provides a potential therapeutic target for early-onset FGR.

## 1 Introduction

FGR refers to the situation where the growth and development of the fetus *in utero* fails to reach its genetic potential due to the influence of pathological factors (Gordijn et al., 2016). It is usually manifested as the estimated weight or abdominal circumference of the fetus by ultrasound being lower than the 10th percentile of the corresponding gestational age (American College of Obstetricians and Gynecologists' Committee on Practice Bulletins—Obstetrics and the Society forMaternal-FetalMedicin, 2019; WHO Alliance for Maternal and Newborn Health Improvement Late Pregnancy Dating Study Group, 2020). As a common pregnancy complication, FGR affects approximately 10% of pregnancies (Nardozza et al., 2017). Particularly the early-onset subtype, is a severe pregnancy complication associated with significant perinatal morbidity and long-term cardiovascular and metabolic risks for the offspring (Crispi et al., 2018; Gordijn et al., 2018). The pathogenesis of FGR remains complex and is not yet fully understood. The placenta, as a key organ for fetal growth and development, plays an irreplaceable role in oxygen supply, nutrient transportation and metabolic waste excretion. Its functional abnormalities are considered a primary factor in FGR pathogenesis (Salafia et al., 1995). Placental dysfunction in FGR is primarily driven by defective spiral artery remodeling and impaired villous development (Burton and Jauniaux, 2018; Dall'Asta et al., 2023). Successful placenta implantation depends on precise spatiotemporal coordination between invasive trophoblastic differentiation and establishment of an immunotolerant microenvironment (Turco and Moffett, 2019). In FGR, this balance is disrupted, leading to characteristic shallow trophoblast invasion and a sterile inflammatory state (Kim et al., 2008). However, the specific cellular drivers and molecular networks regulating this pathological disruption remain poorly understood (Brosens et al., 2011). Therefore, in-depth exploration of gene expression profiles in placental dysfunction is of vital importance to reveal the molecular mechanism of FGR pathogenesis and to develop more effective prevention and treatment strategies.

The physiological transformation of the uterine spiral arteries requires that the EVT undergo a complex differentiation process. These cells originate from the columnar cells of the anchoring villi. Villous Cytotrophoblasts (VCT) penetrate the Syncytiotrophoblasts (SCT), migrate into the decidua, and differentiate into the interstitial EVT (iEVT) and endovascular EVT (enEVT). These special cells anchor the placenta and remodel the maternal blood vessels by replacing the resident endothelial lining, thereby establishing a low resistance circulation (Arutyunyan et al., 2023). It is crucial that this invasive and endothelial layer replacement process is strictly regulated by the local microenvironment. Although early development requires a physiological hypoxic environment, in FGR, persistent hypoxic stress may affect the differentiation of trophoblast cells, inhibit the invasion of EVT and lead to insufficient placental perfusion (Stanek, 2013; Zang et al., 2024)^,^. This delicate balance is regulated by the placental immune microenvironment. Hofbauer cells (HBC), as the only resident macrophages in the fetal placenta, are key regulators of tissue remodeling, angiogenesis, and immune tolerance (Baráth et al., 2025). Although changes in HBC are related to placental inflammation, how different types of HBC interact with trophoblast cells and endothelial cells to trigger the pathogenesis of fetal growth restriction remains a key unknown area.

To address these challenges, we used snRNA-seq to construct a high-resolution transcriptomic atlas of the human placenta in patients with early-onset FGR (Vento-Tormo et al., 2018). Our analysis reveals dysregulation in both trophoblastic and immune cells within FGR. Specifically, we found a loss of a pro-invasive transition state in the trophoblasts of FGR. We also resolved a specific Hofbauer cell subset (HBC5) characterized by high FOS/NFKB1 regulon activity, which is increased in FGR. Further studies showed that HBC5 not only deprived trophoblast cells of essential nutritional support, but also triggered inflammation and metabolic blockade. These findings establish a molecular framework for immune driven placental dysplasia and highlight the potential of AP-1/NF-κB signaling axis and HBC5 subset as therapeutic targets for early-onset FGR.

## 2 Methods

### 2.1 Patient cohort and clinical characteristics

The study received ethical approval from the institutional review board of Nanfang Hospital. Informed written consent was obtained from all individuals. Placental tissues were collected from the Department of Obstetrics and Gynecology at Nanfang Hospital between July 2023 and December 2023. The cohort consisted of three cases of early-onset fetal growth restriction (FGR) and three pregnancies with normal controls (NC). The FGR diagnosis was established in accordance with the consensus-based definition from the International Society of Ultrasound in Obstetrics and Gynecology (ISUOG) (Gordijn et al., 2016; American College of Obstetricians and Gynecologists' Committee on Practice Bulletins—Obstetrics and the Society forMaternal-FetalMedicin, 2019). Pregnancies with multiple gestations, or with diagnosed fetal genetic or congenital abnormalities, were excluded from the study. None of the participants experienced spontaneous labor prior to cesarean section. All participants provided written informed consent, and this study has received ethical approval from the Medical Ethics Committee of Nanfang Hospital, Southern Medical University (approval number: NFEC-202109-K15).

### 2.2 Single-nucleus isolation

Nuclei were isolated from OCT-embedded frozen placental villous tissue sections using a Nucleus Isolation Kit (SHBIO, 52,009–10) according to the manufacturer’s protocol, with the addition of RNase inhibitors to all solutions. Briefly, tissues were minced, homogenized in chilled lysis buffer, and filtered through a 40 μm cell strainer. Nuclei concentration and integrity were assessed using a Fluorescence Cell Analyzer. Nuclei with intact morphology and minimal cytoplasmic contamination were selected for subsequent library preparation.

### 2.3 Single-nucleus RNA sequencing library construction and sequencing

Single-nucleus RNA sequencing (snRNA-seq) libraries were constructed using the SeekOne® Digital Droplet Single Cell 3′ Library Preparation Kit on the SeekOne® DD Chip S3 platform (SeekGene, China). Isolated nuclei were mixed with reverse transcription master mix and co-encapsulated with uniquely barcoded hydrogel beads within droplets. Post-encapsulation steps, including reverse transcription, cDNA amplification, and final library construction, were performed per the manufacturer’s standard protocol. Pooled libraries were sequenced on an Illumina NovaSeq 6,000 platform.

### 2.4 Data processing

Raw sequencing data were processed as follows: adaptor sequences and low-quality bases were trimmed using fastp (v0.23.1). Cleaned reads were aligned to the human reference genome (GRCh38) and a feature-barcode matrix was generated using the Seeksoul pipeline (SeekGene). Downstream analysis was conducted in R (v4.2.0) using the Seurat package (v4.1.1) (Hao et al., 2021).

### 2.5 Cell clustering and annotation

Following quality control to retain high-quality nuclei (featuring >200 detected genes and <20% mitochondrial transcript content), the gene expression matrices were normalized and scaled. Batch effects across samples were corrected using the Harmony algorithm (v0.1) (Korsunsky et al., 2019). Unsupervised clustering was then performed using Seurat. For cell type annotation, we identified cluster-specific marker genes with the FindAllMarkers function in Seurat. Cell identities were assigned by cross-referencing these markers with established cell-type signatures from published placental single-cell datasets (Tsang et al., 2017; Yang et al., 2021; Zhang et al., 2021; Chen et al., 2022; Li et al., 2022; Zhou et al., 2022; Campbell et al., 2023; Ji et al., 2024; Li et al., 2024) and curated databases, including CellMarker 2.0 (Hu et al., 2023) and The Human Protein Atlas. This multistep strategy ensured robust and biologically relevant annotation of all nuclei clusters.

### 2.6 DEGs and GO enrichment analysis

Differentially expressed genes (DEGs) between FGR and NC groups within each cell type were identified using the FindAllMarkers function in Seurat, with thresholds set at an adjusted *p*-value < 0.05, |log2FC| > 1.5, and *p*-value < 0.05. Gene Ontology (GO) and Reactome pathway over-representation analysis (ORA) were performed on the significant DEGs using the clusterProfiler R package. A false discovery rate (FDR) correction was applied to account for multiple testing, and only terms with an FDR (*q*-value) < 0.05 were considered statistically significant.

### 2.7 Pseudotime analysis

We inferred the pseudotime trajectory of trophoblasts with R package Monocle 2 (v2.26.0) (Trapnell et al., 2014). The Differential Gene Test function was employed to detect genes significant to respective cell subsets. After reducing dimensions and sorting cells using default settings, the developmental path was reconstructed.

### 2.8 Intercellular communication analysis

Cell-cell communication between different cells were inferred using the R package CellChat (v1.6.1). The analysis was performed on normalized expression data using the built-in CellChatDB human ligand-receptor interaction database (Jin et al., 2021). Communication probabilities were calculated, and differential interaction strength between the FGR and NC groups was compared to identify significantly altered signaling pathways.

### 2.9 Multiplex immunofluorescence staining (mIF)

Formalin-fixed paraffin-embedded (FFPE) placental sections (4 μm) were deparaffinized and rehydrated. Antigen retrieval was performed using EDTA buffer (pH 9.0). To identify the HBC5 subset, sections were incubated with primary antibodies against CD163 (HBC marker), NFKB1, and FOS, followed by incubation with corresponding HRP-conjugated secondary antibodies and tyramide signal amplification (TSA) fluorophores. Nuclei were counterstained with DAPI. The whole slide images were captured using an Olympus VS200 research slide scanner (Olympus, Tokyo, Japan). For quantification, 5–10 random fields at ×400 magnification were captured per section. The number of DAPI^+^ total nuclei, CD163^+^ cells, FOS^+^ cells, NFKB1^+^ cells, and CD163^+^FOS^+^NFKB1^+^ triple-positive cells were counted using QuPath software (v0.6.0). The percentage of triple-positive cells relative to total DAPI^+^ nuclei was calculated for each sample, and group comparisons were performed using Student’s t-test.

### 2.10 Cell culture and *in vitro* hypoxia induction

The human monocytic cell line THP-1 and the trophoblast cell line HTR8/SVneo were cultured in RPMI 1640 media, respectively, supplemented with 10% fetal bovine serum (FBS). THP-1 cells were differentiated into macrophages by treatment with 100 nM phorbol 12-myristate 13-acetate (PMA) for 48 h. To simulate the hypoxic microenvironment of FGR, macrophages were treated with varying concentrations of CoCl_2_ (Aladdin, C118624) 0, 50, and 100 μM for 24 h.

### 2.11 Conditioned media collection and trophoblast stimulation

Following CoCl_2_ treatment, the macrophage culture supernatant was discarded, and cells were washed and replenished with fresh serum-free medium for an additional 24 h. This conditioned media (CM) was then collected and centrifuged to remove cell debris. HTR8/SVneo cells were incubated with these macrophage-derived CM for 24 h to assess paracrine effects.

### 2.12 Recombinant protein stimulation

To evaluate the specific effects of HBC5-secreted ligands, HTR8/SVneo cells were treated with recombinant human CCL4 (MCE, HY-P7257) or CCL3L1 (MCE, HY-P7231) at concentrations of 0, 50, and 100 ng/mL for 24 h. After stimulation, cells were harvested for RNA extraction and functional gene analysis.

### 2.13 RNA extraction and quantitative real-time PCR (RT-qPCR)

Total RNA was extracted from cells using TRIzol reagent (Invitrogen). cDNA was synthesized using a reverse transcription master mix (TaKaRa, RR047 A). Quantitative real-time PCR (RT-qPCR) was performed using SYBR Green Master Mix (Yeason, 11201 ES) on a Roche LightCycler 96 System (Roche Diagnostics, Basel, Switzerland). The relative mRNA expression levels of target genes *HIF1A*, *FOS*, *NFKB1*, *CCL4*, *CCL3L1*, *CCL4L2*, *NAMPT*, *GDF15* and *APP* were calculated using the 2^−ΔΔCt^ method, with ACTB as the internal control. Primer sequences are provided in Supplementary Table.

### 2.14 Transwell invasion assay

HTR8/SVneo cells were serum-starved overnight, then 5 × 10^4^ cells in 200 μL serum-free medium were seeded into the upper chamber of Transwell inserts (8 μm pore size, Corning) pre-coated with 50 μL Matrigel (BD Biosciences, 1:8 dilution). The lower chamber contained 600 μL RPMI 1640 with 10% FBS as a chemoattractant. Recombinant human CCL4 (MCE, HY-P7257) was added to the upper chamber at 0 or 100 ng/mL. After 24 h of incubation, non-invading cells on the upper membrane surface were removed with a cotton swab. Invaded cells on the lower surface were fixed with 4% paraformaldehyde, stained with 0.1% crystal violet, and photographed. Five random fields per insert were counted, and the average number of invaded cells per field was calculated. Three independent experiments were performed.

### 2.15 Statistical analysis

Data are presented as mean ± standard deviation (SD) from three independent experiments. Statistical analyses and data visualization were performed using R software (v 4.2.2). Differences between two groups were evaluated by two-tailed Student’s t-test, while differences among multiple groups were evaluated by one-way analysis of variance (ANOVA), followed by Tukey’s *post hoc* test for pairwise comparisons using the multcomp package. *P* < 0.05 was considered statistically significant (*P < 0.05, **P < 0.01, ***P < 0.001, ****P < 0.0001; ns, not significant). For differential expression analysis (FindAllMarkers), *P* values were adjusted using Bonferroni correction. For GO enrichment, FDR (Benjamini–Hochberg) correction was applied with a significance threshold of q < 0.05. For Monocle 2 pseudotime analysis, a qval < 0.01 was used to identify genes with dynamic expression. For CellChat, Benjamini–Hochberg correction was applied to the permutation-based communication probabilities.

## 3 Results

### 3.1 Single-nucleus transcriptomic atlas of term FGR and normal placentas

**FIGURE 1:**
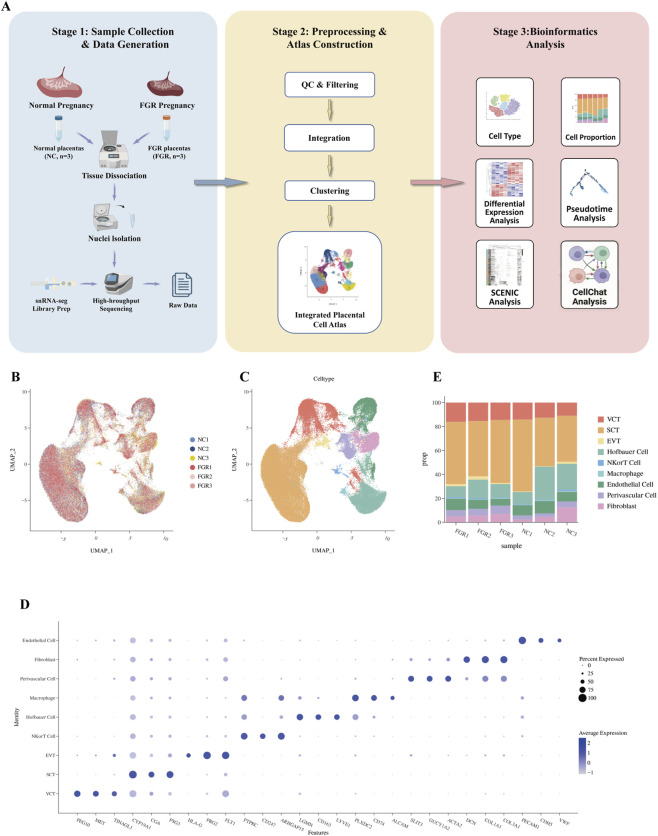
Construction of the single-nucleus transcriptomic atlas of human placenta in Fetal Growth Restriction (FGR) and Normal Control (NC) pregnancies. **(A)** Schematic overview of the experimental workflow. Placental tissue samples were collected from normal pregnancies (n = 3) and pregnancies complicated by FGR (n = 3). Nuclei were isolated for snRNA-seq library preparation and high-throughput sequencing. The bioinformatics pipeline included quality control (QC), data integration, clustering, and downstream analyses. **(B)** UMAP visualization of the integrated dataset containing nuclei from all six samples, colored by group identity. **(C)** UMAP projection of the major cell types identified in the placental atlas. **(D)** Dot plot displaying the expression of canonical marker genes used for cell type annotation. The size of the dot represents the percentage of nuclei expressing the gene, while the color intensity indicates the average expression level. **(E)** Stacked bar plot showing the relative proportions of each cell type across individual samples in the FGR and NC groups.

To dissect the cellular heterogeneity underlying fetal growth restriction (FGR), we performed single-nucleus RNA sequencing (snRNA-seq) on placental tissues from three early-onset FGR cases and three matched healthy controls (Figure 1A). Detailed clinical characteristics of the study cohort are provided in Supplementary Table. Following stringent quality control (Supplementary Figure S1A), a final dataset of 90,852 high-quality nuclei was obtained. Data integration effectively removed batch effects, as evidenced by the homogeneous mixing of nuclei from both conditions within clusters in the integrated UMAP space, indicating minimal technical bias (Figure 1B). Unsupervised clustering identified major placental cell lineages (Figure 1C). We annotated these clusters based on the expression of canonical marker genes established in the literature. Specifically, trophoblast lineages were identified by *PEG10* (Villous Cytotrophoblasts, VCT), *CGA* (Syncytiotrophoblasts, SCT), and *HLA-G* (Extravillous trophoblasts, EVT). Crucially, we clearly identified the resident fetal macrophages, Hofbauer cells (HBC), which formed a distinct cluster characterized by high expression of *LGMN, CD163*, and *LYVE1*. Other stromal components, including endothelial cells (*PECAM1*) and fibroblasts (*COL1A1*), were also clearly resolved. A dot plot summarizes the specificity of these markers across clusters (Tsang et al., 2017; Yang et al., 2021; Zhang et al., 2021; Chen et al., 2022; Li et al., 2022; Zhou et al., 2022; Campbell et al., 2023; Ji et al., 2024; Li et al., 2024) (Figure 1D). Quantification of cell type proportions revealed that while some inter-individual heterogeneity existed, the overall composition of major lineages was broadly comparable between FGR and control groups (Figure 1E).

### 3.2 Abnormal differentiation trajectory and dysfunction of trophoblast cells in FGR

**FIGURE 2:**
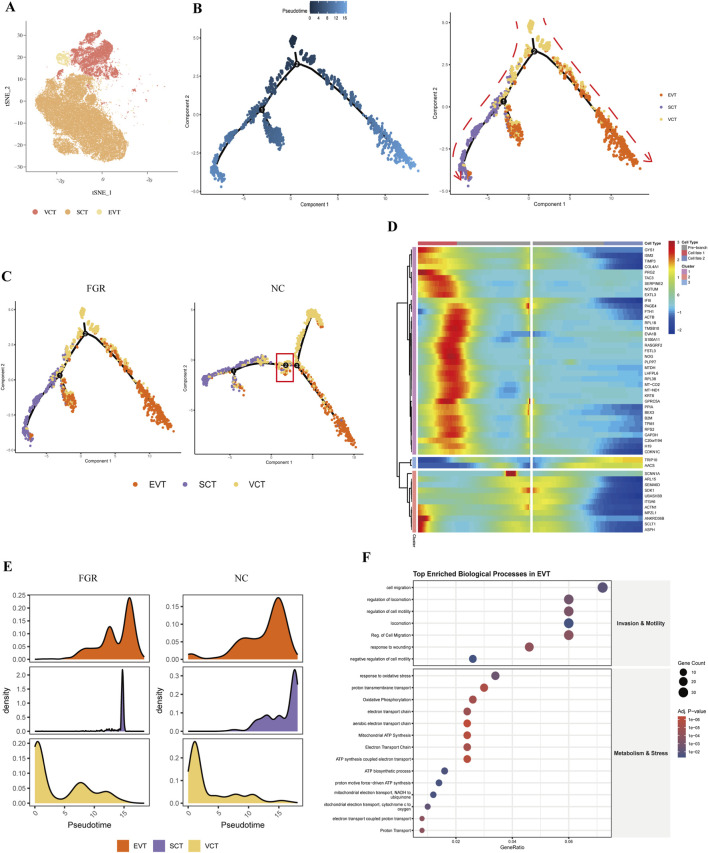
Pseudotime trajectory of trophoblast lineage in FGR and NC placentas. **(A)** t-SNE visualization of the three major trophoblast subpopulations: Villous Cytotrophoblasts (VCT), Syncytiotrophoblasts (SCT), and Extravillous Trophoblasts (EVT). **(B)** Pseudotime trajectory analysis inferring the differentiation lineage of trophoblasts. Left: Cells colored by pseudotime (dark to light blue indicating progression). Right: Cells colored by cell type, showing the bifurcation of VCT into SCT and EVT lineages. **(C)** Comparative trajectory plots split by condition (left: FGR; right: NC). A distinct pro-invasive branch (indicated by a red box) is clearly observed in the NC group but is nearly completely absent in the FGR group. **(D)** Branched heatmap showing the dynamic expression patterns of pseudotime-dependent genes. Rows represent genes and columns represent cells ordered by pseudotime. Genes are clustered into modules based on their expression kinetics during the cell fate specification (Cell fate 1 vs. Cell fate 2). **(E)** Density plots displaying the distribution of VCT, SCT, and EVT along the pseudotime axis in FGR and NC groups. **(F)** Dot plot of the Gene Ontology Biological Process (GOBP) enrichment analysis for downregulated genes in the EVTs of the FGR.

We next analyzed the developmental trajectory of trophoblasts, which are primarily composed of VCT, SCT, and EVT (Figure 2A). Pseudotime analysis of all samples using Monocle 2 revealed a trajectory consistent with the conventional view of differentiation from VCT into SCT and EVT (Okae et al., 2018) (Figure 2B). However, when we analyzed the FGR group and the normal control (NC) group separately, we found that the NC had a branch, while this branch was almost completely absent in the FGR (Figure 2C). The absence of the pro-invasive branch in FGR was observed in all three individual FGR samples when analyzed separately (Supplementary Figure S3A-B). To analyze the molecular characteristics of this state, we examined the dynamic gene expression (Figure 2D). The characteristic feature of this unique state is the upregulation of the invadopodia regulator *TRIP10* and *AACS*, while accompanied by the downregulation of the basement membrane markers *ITGA6* and *COL4A1*, as well as the tissue inhibitory factor *TIMP3*. These molecular features suggest that this branch represents a critical pro-invasive remodeling state, in which trophoblasts detach from the basement membrane and acquire migratory capacity. However, FGR trophoblast cells do not have this branch.

Abnormal differentiation, invasion and vascular remodeling of EVT are the key pathological features underlying FGR (Kaufmann et al., 2003). However, the specific mechanism of dysregulated differentiation has not been fully elucidated. Our pseudotime analysis showed that FGR trophoblasts exhibited a premature peak in SCT differentiation but a delayed progression in EVT differentiation (Figure 2E). Consistent with previous studies, the premature differentiation of SCT may lead to premature maturation of placental barrier function and impaired nutrient exchange capacity (Redline, 2015). The delayed differentiation of EVT may damage the remodeling of uterine spiral arteries and aggravate placental ischemia and hypoxia (Burton and Jauniaux, 2018).

In addition to disturbed differentiation, we sought to determine whether the function of EVT of FGR was impaired. We further performed the Gene Ontology Biological Process (GOBP) analysis of downregulated genes in EVT of FGR placentas. The results showed that these genes were significantly enriched in pathways related to cell migration and motility. Moreover, the analysis highlighted impairments in metabolic pathways, including oxidative phosphorylation, electron transport chain, and mitochondrial ATP synthesis (Figure 2F). Given that trophoblast invasion is an energy demanding process, inadequate energy metabolism may be responsible for the motility defects. Together, these results suggest that EVT in FGR not only has a delayed differentiation process, but also has severe defects in critical functions of invasion, migration and energy metabolism.

### 3.3 FGR exhibits a proinflammatory microenvironment characterized by elevated inflammatory mediators

**FIGURE 3:**
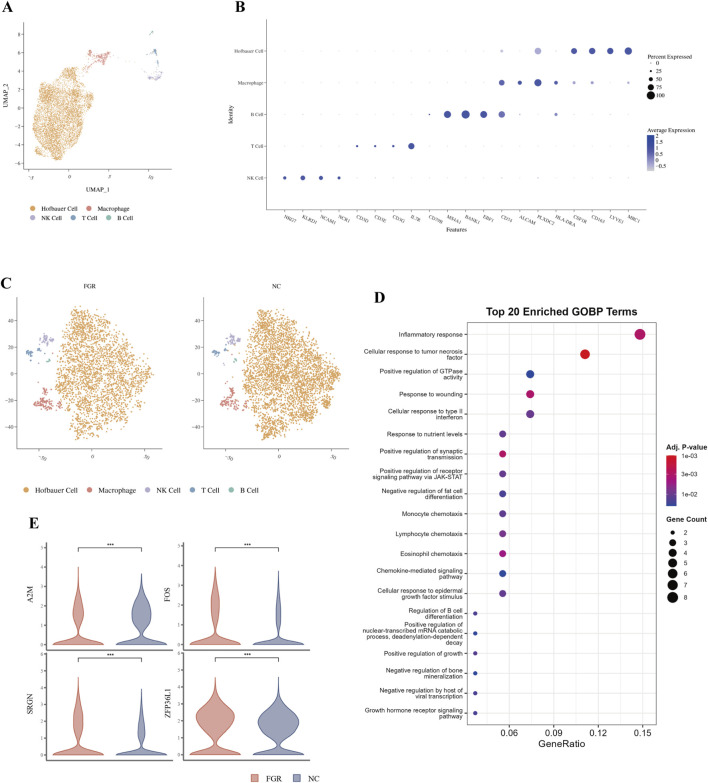
Distinct immune landscape and inflammatory activation in FGR placentas. **(A)** UMAP visualization of the re-clustered immune cell compartment. Five major clusters were identified: Hofbauer cells, Macrophages, NK cells, T cells, and B cells. **(B)** Dot plot showing the expression of canonical marker genes used to annotate each immune cell type. The size of the dot reflects the percentage of cells expressing the gene, and the color intensity indicates the average expression level. **(C)** Split t-SNE plots comparing the distribution of immune cell populations between FGR (left) and NC (right) groups. **(D)** Dot plot of the top 20 enriched Gene Ontology Biological Process (GOBP) terms based on differentially expressed genes (DEGs) in the immune compartment. **(E)** Violin plots illustrating the differential expression of key inflammation-associated genes (*A2M, FOS, SRGN*, and *ZFP36L1*) between FGR and NC groups. Statistical significance is indicated (*P* < 0.001).

To investigate the immune microenvironment in FGR, we sub-clustered the immune cell populations from the integrated dataset and identified five distinct cell types: Hofbauer cells (HBC), macrophages, natural killer (NK) cells, T Cells, and B cells (Figure 3A). Their identities were validated by characteristic lineage markers: *CD163* and *LYVE1* for HBC, *ALCAM* and *PLXDC2* for macrophages, *MS4A1* and *BANK1* for B cells, *CD3D* and *CD3E* for T Cells, and *NKG7* and *KLRD1* for NK cells (Figure 3B).

Although the overall cellular composition remained comparable between FGR and control (Figure 3C), differential gene expression analysis revealed substantial functional alterations. The GO enrichment analysis of the upregulated genes in FGR immune cells showed that pathways related to inflammatory response, chemotaxis, and response to wounding were significantly enriched (Figure 3D), indicating that immune dysregulation in FGR is primarily qualitative rather than quantitative.

This functional shift is manifested at the molecular level by extensive stress response and upregulation of proinflammatory mediators. Specifically, the stress-inducible transcription factor *FOS* and the mRNA binding regulator *ZFP36L1* were significantly elevated. Meanwhile, genes encoding key inflammatory mediators, acute-phase response protein *A2M*, and the heparan sulfate proteoglycan *SRGN*, which regulates granule secretion, were upregulated in immune cells (Figure 3E). Collectively, these molecular features point to a chronic, noninfectious inflammatory state in the placental immune microenvironment of FGR.

### 3.4 Hypoxia-driven transcriptional reprogramming defines the proinflammatory HBC5 subset

**FIGURE 4:**
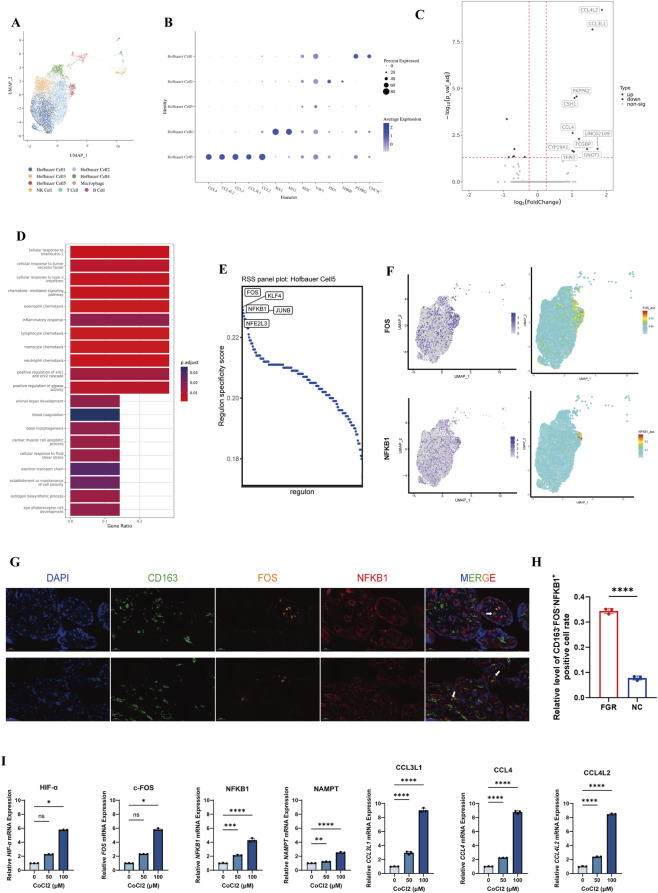
Transcriptional heterogeneity and regulatory network analysis of Hofbauer cell subsets. **(A)** UMAP visualization of the immune cell compartment with high-resolution subclustering of Hofbauer cells. Five distinct subsets (Hofbauer Cell 1–5) were identified alongside other immune lineages. **(B)** Dot plot displaying the expression of marker genes for each Hofbauer cell subset. **(C)** Volcano plot illustrating the differentially expressed genes (DEGs) between FGR and NC groups within the Hofbauer Cell 5 subset. Red dots represent significantly upregulated genes, and blue dots represent significantly downregulated genes. **(D)** Bar plot showing the top enriched Gene Ontology Biological Process (GOBP) terms for the differentially expressed genes in the Hofbauer Cell 5 subset (FGR vs. NC), colored by adjusted p-value. **(E)** Regulon Specificity Score (RSS) plot for Hofbauer Cell 5 derived from SCENIC analysis. **(F)** UMAP projection showing the correspondence between gene expression (left panels) and regulon activity (AUC scores, right panels) for key transcription factors FOS and NFKB1. **(G)** The representative multiplex immunofluorescence (mIF) images show the expression of CD163 (green), FOS (yellow), and NFKB1 (red) in the FGR placental tissue. White arrows indicate the CD163+FOS + NFKB1+ triple-positive HBC5 cells in the FGR group. The scale bar = 25 μm. **(H)** Quantification of CD163^+^FOS^+^NFKB1^+^ triple-positive cells as a percentage of total DAPI^+^ nuclei in FGR (n = 3) and NC (n = 3) placentas from an independent mIF cohort. Data are mean ± SD. ****P < 0.0001 (Student’s t-test). **(I)** RT-qPCR was used to verify the THP-1 macrophages induced by the treatment with CoCl_2_ solutions of concentrations of 0, 50 and 100 μM. The mRNA expression levels of hypoxia markers (*HIF1A*), master transcription factors (*NFKB1*, *FOS*), and HBC5 specific effector factors (*CCL3L1*, *CCL4*, *CCL4L2* and *NAMPT*) were demonstrated. All these markers showed significant dose-dependent upregulation under simulated hypoxic conditions. Data are presented as mean ± SD (n = 3). *P < 0.05, **P < 0.01, ***P < 0.001, ****P < 0.0001 compared with the control group (0 μM).

To clarify the driving factors of inflammation, we performed subgroup analysis of the HBC with the highest number of immune cells, resulting in 5 clusters (HBC1-5) (Figure 4A). Each subclone exhibited unique transcriptional signatures. HBC1 is a subcluster with high expression of *PTPRK*, *CADM2* and *SYT1*, possibly involved in intercellular interactions and tissue structure maintenance. HBC2 is a subcluster with high expression of *CD74*, *HLA-DRA, MSR1*, likely possessing immune surveillance and scavenger functions. HBC3 is a subgroup with high expression such as *SPI1*, *VAV1*, *ARRB1*, possibly being in an intermediate state of immune regulation and activation. HBC4 is a subgroup with high expression of *MX1*, *IFIT1-3*, *ISG15*, possibly in an antiviral response state. HBC5 is a highly proinflammatory subcluster, characterized by strong expression of chemokines (*CCL2*, *CCL3* and *CCL4*) and the expression of metabolic stress regulatory factor *NAMPT* (Figure 4B; Supplementary Figures S1B,S2A).

Previous studies suggest that HBCs in full-term placenta are often classified as anti-inflammatory M2 macrophages (Reyes and Golos, 2018). However, in pathological conditions such as PE, the number of anti-inflammatory HBCs decreases, shifting toward pro-inflammatory subtypes (Mercnik et al., 2022). Similarly, in our study, the HBC5 subcluster is enriched in FGR, accounting for 4.41% of the total HBC, while it was only 2.63% in NC (Supplementary Figure S2B). Differential enrichment analysis of HBC5 revealed that the inflammatory chemokines *CCL4L2*, *CCL3L1*, and *CCL4* were significantly upregulated in FGR (Figure 4C). Notably, CCLs family chemokines are potent inflammatory mediators known to play a central role in inflammatory diseases, exhibiting both chemotactic and pro-inflammatory capabilities (Hannan et al., 2006). GOBP results showed that the HBC5 of FGR became a recruitment center through active chemokine signaling pathways, capable of recruiting almost all major immune cells, such as monocytes/macrophages, lymphocytes, neutrophils, and eosinophils (Figure 4D). We tested multiple resolution parameters (0.1–0.3) during HBC subclustering. At resolution 0.2, HBC5 was not fully separated from neighboring subsets; at resolution 0.3 and above, HBC5 emerged as a distinct cluster characterized by high *CCL4*, *CCL4L2*, *CCL3L1*, *CCL3* and *CCL2* expression (Supplementary Figure S4C).

To investigate potential upstream regulators associated with these HBC5 cells, we used SCENIC analysis to infer the gene regulatory networks. The Regulon Specificity Score (RSS) identified FOS (a core component of AP-1) and NFKB1 as the top ranking regulators specific to HBC5 (Figure 4E). This was further confirmed by UMAP visualization (Figure 4F) and binary activity heatmap (Supplementary Figure S2C), which demonstrated that FOS and NFKB1 regulon activities were preferentially enriched in the HBC5. These data suggest that the inflammatory program involving AP-1/NF-κB regulons is enriched in HBC5.

To verify the existence of this pathogenic cell subset and its spatial distribution, we conducted multiplex immunofluorescence staining on placental tissues. Consistent with our bioinformatics prediction results, we found that CD163^+^ FOS^+^ NFKB1^+^ triple-positive cells were specifically present only in the stromal tissue of the placenta with FGR, although their number was relatively small (Figure 4G). In contrast, in normal control tissues, these cells were almost absent or undetectable. To quantitatively validate the finding that HBC5 is enriched in FGR, we performed multiplex immunofluorescence on an independent cohort of 3 FGR and 3 NC placentas and quantified CD163^+^ FOS^+^ NFKB1^+^ triple-positive cells. The percentage of triple-positive cells among total DAPI^+^ nuclei was significantly higher in FGR placentas (Figure 4H) compared to NC placentas. These quantitative data independently confirm the specific enrichment of the HBC5 subset in early-onset FGR. This unique distribution pattern further suggests that the HBC5 phenotype, which correlates with high FOS/NFKB1 regulon activity, is a specific pathological feature of the microenvironment of fetal growth restriction. Additionally, although HBC5 nuclei were more abundant in male placentas, the male-to-female ratio was comparable between groups, and expression of core signature genes did not differ by fetal sex (Supplementary Figure S4A-B). These data suggest that the HBC5 enrichment observed in FGR is not driven by fetal sex imbalance.

Considering the typical hypoxic microenvironment in the FGR placenta, we further investigated whether environmental stress drives the generation of the specific phenotype of HBC5. We established an *in vitro* model using THP-1 induced macrophages and treated them with different concentrations of CoCl_2_ (0, 50, and 100 μM) for 24 h to simulate different degrees of hypoxia. The RT-qPCR results showed that the mRNA level of the hypoxia marker *HIF1A* increased along with CoCl_2_ concentration, confirming the successful establishment of the *in vitro* hypoxia model. At the same time, the expression levels of core transcription factors *NFKB1*, *FOS*, and HBC5 markers associated with pro-inflammation and metabolism *CCL3L1*, *CCL4*, *CCL4L2*, and *NAMPT* also significantly increased (Figure 4I). These experimental results indicate that the hypoxic microenvironment is a key external factor driving the inflammatory and metabolic remodeling in the HBC5 subset. This discovery firmly links placental hypoxia with the characteristic chronic inflammation in FGR, revealing the pathological role of HBC5 as an environmental stress regulator.

### 3.5 HBC5 rewires communication with EVT and endothelial cells toward a proinflammatory dialogue in FGR

**FIGURE 5:**
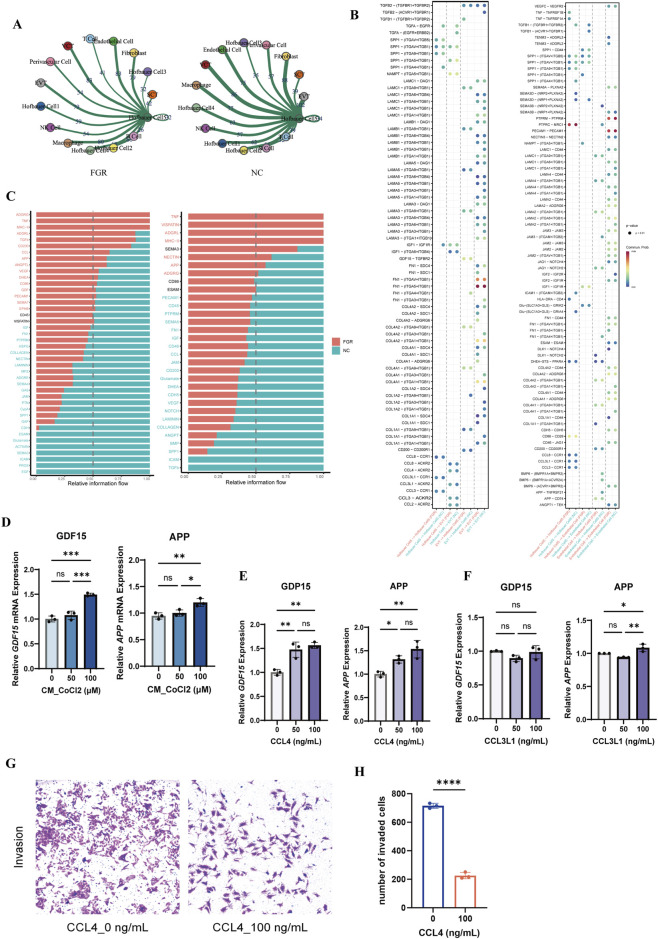
Aberrant crosstalk between the inflammatory Hofbauer Cell 5 subset and placental structural cells in FGR. **(A)** Circle plots illustrating the global cell-cell communication network in FGR (left) and NC (right) placentas. Edge thickness represents interaction strength, and numbers indicate significant ligand-receptor pairs. **(B)** Dot plots visualizing specific ligand-receptor interactions. Left: Interactions between HBC5 and EVT. Right: Interactions between HBC5 and Endothelial Cells. The color scale represents communication probability, and dot size indicates statistical significance. **(C)** Stacked bar plots ranking signaling pathways based on relative information flow differences between FGR (red) and NC (blue). Left: Pathways mediating the crosstalk between HBC5 and EVT. Right: Pathways mediating the crosstalk between HBC5 and Endothelial Cells. **(D–F)** Functional validation of the HBC5-EVT axis. **(D)** RT-qPCR analysis of GDF15 and APP expression in HTR8/SVneo cells treated with conditioned media (CM) from macrophages exposed to 0, 50, and 100 μM CoCl_2_. **(E,F)** mRNA expression levels of GDF15 and APP in HTR8/SVneo cells following stimulation with recombinant CCL4 **(E)** or CCL3L1 **(F)**. **(G,H)** Effect of CCL4 on trophoblast invasion. **(G)** Representative images of Transwell invasion assays of HTR8/SVneo cells treated with 0 or 100 ng/mL recombinant CCL4 for 24 h. Scale bar = 100 μm. **(H)** Number of invaded cells per field. Data are mean ± SD from three independent experiments. **P < 0.01 (Student’s t-test) compared with control (0 ng/mL). Data in **(D–F)** are presented as mean ± SD (n = 3). *P < 0.05, **P < 0.01, ***P < 0.001, ****P < 0.0001, ns, not significant.

To explore the role of HBC5 in the FGR placental microenvironment, we analyzed its intercellular communication network using CellChat. The results showed that in FGR, HBC5 had the strongest interaction with endothelial cells, VCT, macrophages, EVT, and fibroblasts (Figure 5A). Given that the dysfunction of trophoblasts and endothelial cells is central to the pathogenesis of FGR, we then focused on the changes in the communication between HBC5 and these two types of cells. We visualized the abnormal interaction between HBC5 and EVT (Figure 5B, left). In the normal placentas, HBC5 provides growth signals through the IGF1-IGF1R, TGFA-EGFR, and SPP1-ITGAV axes. However, in FGR, most of these signals are lost. Instead, in FGR, HBC5 acts on EVT with strong proinflammatory chemokines CCL2/3/4-ACKR2 and metabolic stress signals NAMPT-integrin. It is important to note that ACKR2 is an atypical decoy receptor that typically scavenges and degrades chemokines rather than transducing signals; therefore, the CCL4-induced trophoblast stress is likely mediated through alternative receptors such as CCR5 or CCR1. In the reverse direction of communication, the EVT in FGR actively transmit stress signals to HBC5 through the upregulation of mitochondrial stress factor *GDF15* and inflammatory mediator *APP* in the feedback loop. However, they failed to provide basement membrane support by downregulating *LAMA3/5*. Consistent with these ligand-receptor changes, the information flow analysis confirmed the functional shift (Figure 5C, left side). The pathways controlling tissue structure, such as laminin, collagen, and insulin-like growth factor, are dominant in the NC. In contrast, the inflammatory and stress pathways, such as tumor necrosis factor (TNF), MHC-II, and ADGRG, are dominant in the HBC5-EVT network of FGR.

Next, we investigated the interaction between HBC5 and endothelial cells (Figure 5B, right side). The normal vascular microenvironment is maintained by signaling pathways such as VEGF, BMP, and NOTCH. However, in FGR, HBC5 exerts metabolic stress on endothelial cells through the NAMPT-(ITGA5 + ITGB1) axis and promotes pathological remodeling through the SPP1-integrin interaction. Conversely, FGR endothelial cells target HBC5 through APP-CD74 and DLK1-NOTCH2 signaling, amplifying the inflammatory circuit. The analysis of information flow (Figure 5C, right side) further confirmed the transformation from steady-state signals such as BMP, CDH5, and VEGF to immunopathological features rich in VISFATIN (NAMPT), APP, and tumor necrosis factor. These data suggest that the proinflammatory HBC5 in FGR may directly contribute to placental dysfunction by rewiring the communication network with EVT and endothelial cells to switch homeostatic growth supporting signals to a sustained inflammatory versus stress dialogue.

To verify whether HBC5-related factors cause trophoblast cell dysfunction, we conducted an *in vitro* stimulation experiment using HTR8/SVneo cells. Firstly, we collected CM from THP-1-derived macrophages exposed to different concentrations (0, 50, and 100 μM) of CoCl_2_ gradient to simulate the hypoxic secretion pattern. RT-qPCR analysis showed that HTR8/SVneo cells treated with hypoxic macrophage CM induced dose-dependent upregulation of *GDF15* and *APP* (Figure 5D). To determine the specific ligand responsible for this effect, we treated HTR8/SVneo cells with recombinant proteins. Treatment with recombinant CCL4 significantly increased the mRNA levels of *GDF15* and *APP* (Figure 5E). In contrast, treatment with recombinant CCL3L1 did not significantly change the expression of *GDF15*, but a moderate increase in *APP* was observed (Figure 5F). These findings indicate that CCL4 is a potent driver of stress experienced by trophoblast cells in the FGR microenvironment, suggesting that the hypoxia-induced inflammatory mediators produced by HBC5 contribute to the disruption of the placental microenvironment. We further investigated whether CCL4 directly affects trophoblast invasive capacity using Transwell invasion assays. Treatment of HTR8/SVneo cells with 100 ng/mL recombinant CCL4 significantly reduced the number of invading cells compared to control (0 ng/mL) (Figures 5G,H). It should be noted that most of the ligand-receptor interactions inferred by CellChat are computational predictions. However, the functional relevance of CCL4 in inducing trophoblast stress is supported by recombinant CCL4 experiments (Figure 5E), and the transmission of stress signals GDF15 and APP from EVT to HBC5 is supported by conditioned media experiments (Figure 5D).

## 4 Discussion

This study constructed snRNA-seq profiles of early-onset FGR and normal placentas, revealing disrupted trophoblast differentiation trajectories and the absence of key invasive trophoblast states in FGR placentas. Additionally, in the placental immune microenvironment, a specific proinflammatory Hofbauer cell subset characterized by high FOS/NFKB1 regulon activity was identified. This subset increases in FGR placentas and disrupts placental homeostasis by reshaping intercellular communication.

Normal placental formation relies on the precise differentiation of VCT into SCT and invasive EVT. Although impaired EVT invasion is a well-known characteristic of FGR (Kaufmann et al., 2003), our trajectory analysis provides a more detailed mechanistic insight. In FGR placentas, there is a deficiency of trophoblast cells in the proinvasive transition state. We found that this missing branch in FGR is characterized by the progressive upregulation of the invasive pseudopod regulatory factor *TRIP10* (Pichot et al., 2010), along with the gradually downregulation of basement membrane anchoring proteins *ITGA6* (Damsky et al., 1994) and *COL4A1*. This indicates that the trophoblast cells in FGR fail to execute the key dissociation program required for detachment from the cell column and initiation of invasion (Knöfler et al., 2019). In addition, we also discovered an imbalance in the differentiation sequence. The differentiation of SCT in FGR reaches its peak prematurely and the peak of EVT differentiation is delayed. This differentiation trajectory may represent a compensatory but maladaptive response of the placenta to stress. The placenta prioritizes maintaining the nutritional transport function of SCT while sacrificing the vascular remodeling function of EVT. Invasion requires a lot of energy. However, in the EVT of FGR, there is a significant downregulation of the oxidative phosphorylation and mitochondrial ATP synthesis pathways. This suggests that even if EVT undergoes differentiation, they may lack the biological energy capacity required to execute migration and vascular remodeling. In conclusion, these findings indicate that there are many common defects in FGR that collectively lead to impaired invasiveness of trophoblast cells.

Although the proinflammatory transformation of the FGR placental microenvironment is a recognized feature (Burton and Jauniaux, 2018), its specific regulatory mechanisms remain unclear. Our data confirm this widespread inflammatory state, manifested by the upregulation of stress response mediators such as *A2M*, *SRGN*, and *FOS* in immune cells. The identification of HBC5 challenges the traditional view of Hofbauer cells as a homogeneous, immunotolerant M2-like macrophage population (Reyes and Golos, 2018). Instead, our findings reveal a special subset within the fetal macrophage lineage.

To our knowledge, no public single-cell or single-nucleus dataset of early-onset FGR is currently available for direct comparison. However, we compared our HBC5 signature with several published placental macrophage datasets from both healthy and complicated pregnancies. In healthy term placentas, Baráth et al. reported that Hofbauer cells exhibit a primarily tolerogenic transcriptional program, and a cluster with high *CCL4*, *CCL3L1* and *NAMPT* similar to our HBC5 was not observed (Baráth et al., 2025). In pregravid obesity, HBCs show increased inflammatory gene expression, but the specific combination of *CCL* chemokines and *NAMPT* observed in HBC5 was not reported as a distinct cluster (Doratt et al., 2025). In preeclampsia, Chen et al. demonstrated that Hofbauer cells and trophoblasts undergo subtype-specific transcriptional and metabolic remodeling (Chen et al., 2026). Using a cross-species approach, Wang et al. showed that in FGR fetal pigs, Hofbauer cells exhibit enhanced inflammation and hypoxia response (Wang et al., 2026). Finally, Hoo et al. reported that infection of early human placental explants triggers pathogen-specific inflammatory responses in Hofbauer cells (Hoo et al., 2024). Taken together, these comparisons support that HBC5 is not a constitutive homeostatic population but likely emerges under the unique hypoxic or inflammatory environment of early-onset FGR.

The global pro-inflammatory immune landscape in FGR is characterized by upregulation of stress-response genes across multiple immune cell types. However, the HBC5 subset exhibits a unique and more acute inflammatory signature, including high expression of *CCL* chemokines and *NAMPT*, which is not observed in other immune populations. HBC5 is therefore likely a key contributor to the inflammatory milieu in the villous stroma, but not the sole orchestrator. Other immune cells may also participate in the sterile inflammatory response.

Mechanistically, SCENIC analysis predicted a transcriptional network in HBC5, with FOS (AP-1) and NFKB1 among the top enriched regulators (Dorrington and Fraser, 2019). This indicates that the increase in HBC5 in the FGR placenta is not a random event but a coordinated response to placental stress. The *in vitro* validation using macrophages treated with CoCl_2_ further supported this model, demonstrating that the hypoxia gradient directly promotes the upregulation of *HIF1A* as well as the key transcription factors *FOS* and *NFKB1*. This confirmed that the polarization of HBC5 is a direct response to the hypoxia microenvironment typically found in FGR. We propose that the upstream process may involve the integration of stress signals such as hypoxia or oxidative stress through the AP-1/NF-κB signaling axis, thereby reprogramming the quiescent Hofbauer cells into effective mediators of sterile inflammation. This suggests that HBC5 may act not only as a participant but also as a key coordinator of placental immune dysregulation. Furthermore, we found that HBC5 in FGR highly expresses chemokines such as *CCL4L2*, *CCL4*, and *CCL3L1*.

Our observation that recombinant CCL4 (100 ng/mL) inhibits trophoblast invasion (Figure 5G,H) appears at odds with a prior study showing that CCL4 promotes trophoblast migration at lower concentrations (Hannan et al., 2006). However, this apparent discrepancy is resolved upon careful re-evaluation of the effective protein concentrations used. Hannan et al. reported using 100 nM CCL4. Based on recombinant CCL4’s molecular weight (∼7.8 kDa), this equals approximately 780 ng/mL. In our study, we selected a concentration of 100 ng/mL (12.8 nM) based on our dose-response experiment (Figure 5E), where it robustly upregulated stress markers GDF15 and APP. The manufacturer’s datasheet further confirms that our CCL4 is bioactive, as its ED_50_ (2.785 ng/mL) effectively chemoattracts THP-1 cells. Therefore, our study does not reveal a simple dose-dependent dichotomy but rather uncovers a context-dependent functional switch. Under the chemotaxis conditions used by Hannan et al., CCL4 promoted migration, whereas in our invasion assay, which mimics the compromised placental barrier in FGR, the same cytokine significantly impaired trophoblast invasion. This finding highlights that CCL4’s role is highly context-dependent, and in the FGR microenvironment, it may contribute to trophoblast dysfunction.

Our analysis of intercellular communication indicates that under physiological conditions, Hofbauer cells provide essential nutritional support such as *IGF1* (Forbes and Westwood, 2008; Sferruzzi-Perri et al., 2017) and *TGFA* to promote EVT growth and invasion. In FGR, HBC5 withdraws this critical support. Instead, it triggers inflammatory blockade by secreting potent chemokines *CCL2/3/4* and metabolic stress signal *NAMPT* (Dahl et al., 2012). As noted in the Results, ACKR2 is a decoy receptor, so the CCL4-induced trophoblast stress is likely mediated through signaling-competent receptors such as CCR5 or CCR1. Moreover, although the normal vascular microenvironment is maintained by proangiogenic signals such as VEGF and BMP, HBC5 in FGR reprograms this interface toward pathological remodeling. Specifically, it exerts metabolic stress on vascular endothelial cells through the NAMPT-integrin axis, while failing to provide stable angiogenic factors such as VEGF. These findings suggest that HBC5 actively disrupts placental structure by simultaneously withdrawing necessary support and applying metabolic stress.

The observed trophoblast cell defects and immune dysregulation in FGR are independent pathological processes, or are they mechanistically interrelated? Integrating our findings, we propose a vicious cycle model for FGR. We hypothesize that the placenta in FGR may be triggered by hypoxia or metabolic stress, which leads to an inappropriate dialogue between structural cells and the immune system. Specifically, the EVT in FGR upregulated the mitochondrial stress factor *GDF15* (Fujita et al., 2016; Wedel et al., 2023) and the inflammatory mediator *APP*, while the vascular endothelial cells targeted Hofbauer cells through the APP-CD74 axis (Liu et al., 2024). *GDF15* is a known biomarker of mitochondrial dysfunction and cellular stress, and the APP-CD74 signaling pathway is associated with macrophage activation and inflammation. We speculate that these stress derived signals act as upstream triggers, activating the FOS/NFKB1 regulatory elements in Hofbauer cells, driving their polarization to the pathogenic HBC5 phenotype. Once established, HBC5 counters by withdrawing nutritional support and imposing metabolic blockade, thereby further exacerbating trophoblast cell stress and vascular dysfunction. This leads the placenta into a state of chronic inflammation and developmental arrest, hindering physiological repair.

Although this study provides high resolution insights, there are still several limitations. Firstly, the use of snRNA-seq on frozen tissues may underestimate the abundant cytoplasmic transcripts, potentially affecting the quantification of certain secretory factors (Slyper et al., 2020), but it is the optimal choice for archived samples. Secondly, as our samples were collected at delivery, representing the terminal stage of FGR pathology. Therefore, the exact starting time point of HBC5 polarization needs to be clarified in future longitudinal studies. Thirdly, although the THP-1 macrophage line is widely used, it does not fully reproduce the developmental origin and tissue-specific features of primary Hofbauer cells. Since isolation of primary HBC from human term placentae is technically challenging, this makes large-scale mechanistic validation difficult. Future studies using primary HBC or iPSC-derived placental macrophages will be of great value. Moreover, the small sample size (n = 3 in each group) limits the generalizability of the results. However, mIF validation in an independent cohort and the high cell depth mitigated this problem to some extent. Finally, although SCENIC implicates FOS and NFKB1 as key regulons, loss of function studies are needed to obtain evidence of causality. At the therapeutic level, two distinct strategies could be considered: inhibiting HBC5 polarization by targeting the upstream AP-1/NF-κB axis, or blocking its effector CCL4-CCR5 pathway using CCR5 antagonists. Each approach has its own potential benefits and risks, warranting future preclinical testing.

## 5 Conclusion

In summary, this study has produced a high-resolution transcriptome map of the placenta in cases of early-onset FGR, revealing a close correlation between pathogenic immune activation and trophoblast dysfunction. We identified a specific Hofbauer cell subset (HBC5) with high FOS/NFKB1 regulon activity, which may play an important role in the disease. Our experimental validation confirms that the emergence of HBC5 is directly triggered by the hypoxic microenvironment. The data support the model of immune driven placental developmental abnormalities, where HBC5 imposes a dual blow on the placental microenvironment. On the one hand, it deprives EVT of essential nutrient support factors *IGF1.* On the other hand, it simultaneously exerts inflammatory and metabolic blocking effects with CCL chemokines and NAMPT. We further propose a vicious cycle: under stress conditions, trophoblast cells transmit danger signals such as *GDF15* and *APP*, thereby continuously exacerbating this immune maladaptation. These findings not only deepen our understanding of the cellular and molecular mechanisms of FGR but also highlight the value of the AP-1/NF-κB signaling axis and the HBC5 subset as potential therapeutic targets and diagnostic biomarkers, providing a new research direction for this pregnancy complication.

## Data Availability

The datasets presented in this study can be found in online repositories. The names of the repository/repositories and accession number(s) can be found below: https://ngdc.cncb.ac.cn/gsa-human, HRA018355.

## Supplementary material

SUPPLEMENTARY FIGURE S1
**(A)** Violin plots showing the quality control (QC) metrics for the six samples, including the number of detected features (nFeature_RNA), total counts (nCount_RNA), and the percentage of mitochondrial genes (mito). **(B)** Volcano plots illustrating the differential gene expression in each of the five Hofbauer cell subsets (HBC1-5), showing the log2 fold change and the log10 of the adjusted p-value. Red dots indicate significantly upregulated genes, blue dots indicate significantly downregulated genes, and grey dots indicate non-significant genes.

SUPPLEMENTARY FIGURE S2
**(A)** Bar charts displaying the top enriched Gene Ontology (GO) terms for the differentially expressed genes in each of the HBC1-5 subsets. The terms are categorized by molecular function, cellular component, and biological process. **(B)** Pie charts comparing the relative proportions of the five Hofbauer cell subsets between FGR (left) and NC (right). **(C)** Binarized heatmap of regulon activity across different Hofbauer cell subsets.

SUPPLEMENTARY FIGURE S3 Monocle 2 pseudotime trajectory analysis of trophoblast differentiation in individual samples. **(A)** State plots of pseudotime trajectories for all cells from the FGR group (left) and NC group (right), analyzed separately. Colors indicate distinct differentiation states. **(B)** Monocle 2 component plot with cells colored by individual sample identity (FGR1, FGR2, FGR3, NC1, NC2, NC3). Each component corresponds to a distinct cell state.

SUPPLEMENTARY FIGURE S4
**(A)** UMAP visualization of Hofbauer cell subsets colored by fetal sex (female vs. male) in FGR and NC groups, showing no sex-specific segregation of HBC5. **(B)** Pie charts illustrating the proportion of each HBC subset (HBC1–5) stratified by fetal sex and group: top left, FGR female; top right, FGR male; bottom left, NC female; bottom right, NC male. **(C)** UMAP plots of immune cell clustering at different resolution parameters (0.1, 0.2, 0.3). HBC5 emerges as a distinct cluster at resolution ≥0.3. **(D)** Pseudotime trajectory of HBC subsets, with HBC5 positioned at the endpoint of a differentiation path from homeostatic subsets. **(E)** Boxplot of CytoTRACE differentiation scores across HBC1–5, showing intermediate scores for HBC5.

## References

[B1] American College of Obstetricians and Gynecologists' Committee on Practice Bulletins—Obstetrics and the Society forMaternal-FetalMedicin (2019). ACOG practice bulletin no. 204: fetal growth restriction. Obstet. Gynecol. 133 (2), e97–e109. 10.1097/aog.0000000000003070 30681542

[B2] Arutyunyan A. Roberts K. Troulé K. Wong F. C. K. Sheridan M. A. Kats I. (2023). Spatial multiomics map of trophoblast development in early pregnancy. Nature 616 (7955), 143–151. 10.1038/s41586-023-05869-0 36991123 PMC10076224

[B3] Baráth B. R. Bojcsuk D. Bene K. Caballero-Sánchez N. Cseh T. de Freitas J. C. (2025). Distinct transcriptional and epigenomic programs define hofbauer cells in term placenta. JCI Insight 11, e195801. 10.1172/jci.insight.195801 41433105 PMC12892902

[B4] Brosens I. Pijnenborg R. Vercruysse L. Romero R. (2011). The “Great Obstetrical Syndromes” are associated with disorders of deep placentation. Am. J. Obstet. Gynecol. 204 (3), 193–201. 10.1016/j.ajog.2010.08.009 21094932 PMC3369813

[B5] Burton G. J. Jauniaux E. (2018). Pathophysiology of placental-derived fetal growth restriction. Am. J. Obstet. Gynecol. 218 (2), S745–s761. 10.1016/j.ajog.2017.11.577 29422210

[B6] Campbell K. A. Colacino J. A. Puttabyatappa M. Dou J. F. Elkin E. R. Hammoud S. S. (2023). Placental cell type deconvolution reveals that cell proportions drive preeclampsia gene expression differences. Commun. Biol. 6 (1), 264. 10.1038/s42003-023-04623-6 36914823 PMC10011423

[B7] Chen J. Du L. Wang F. Shao X. Wang X. Yu W. (2022). Cellular and molecular atlas of the placenta from a COVID-19 pregnant woman infected at midgestation highlights the defective impacts on foetal health. Cell Prolif. 55 (4), e13204. 10.1111/cpr.13204 35141964 PMC9055894

[B8] Chen Y. X. Wu L. L. Huang D. N. Wu X. X. Liu K. Sun B. (2026). Subtype specific immune-metabolic reprogramming in preeclampsia revealed by multiomics and serum biomarkers. Hypertens. Res. 49 (3), 641–657. 10.1038/s41440-025-02504-5 41419624 PMC12960252

[B9] Crispi F. Miranda J. Gratacós E. (2018). Long-term cardiovascular consequences of fetal growth restriction: biology, clinical implications, and opportunities for prevention of adult disease. Am. J. Obstet. Gynecol. 218 (2), S869–s879. 10.1016/j.ajog.2017.12.012 29422215

[B10] Dahl T. B. Holm S. Aukrust P. Halvorsen B. (2012). Visfatin/NAMPT: a multifaceted molecule with diverse roles in physiology and pathophysiology. Annu. Rev. Nutr. 32, 229–243. 10.1146/annurev-nutr-071811-150746 22462624

[B11] Dall'Asta A. Melito C. Morganelli G. Lees C. Ghi T. (2023). Determinants of placental insufficiency in fetal growth restriction. Ultrasound Obstet. Gynecol. 61 (2), 152–157. 10.1002/uog.26111 36349884

[B12] Damsky C. H. Librach C. Lim K. H. Fitzgerald M. L. McMaster M. T. Janatpour M. (1994). Integrin switching regulates normal trophoblast invasion. Development 120 (12), 3657–3666. 10.1242/dev.120.12.3657 7529679

[B13] Doratt B. M. True H. E. Sureshchandra S. Qiao Q. Rincon M. Marshall N. E. (2025). The immune landscape of fetal chorionic villous tissue in term placenta. Front. Immunol. 15, 18. 10.3389/fimmu.2024.1506305 39872537 PMC11769816

[B14] Dorrington M. G. Fraser I. D. C. (2019). NF-κB signaling in macrophages: dynamics, crosstalk, and signal integration. Front. Immunol. 10, 705. 10.3389/fimmu.2019.00705 31024544 PMC6465568

[B15] Forbes K. Westwood M. (2008). The IGF axis and placental function. A mini review. Horm. Res. 69 (3), 129–137. 10.1159/000112585 18219215

[B16] Fujita Y. Taniguchi Y. Shinkai S. Tanaka M. Ito M. (2016). Secreted growth differentiation factor 15 as a potential biomarker for mitochondrial dysfunctions in aging and age-related disorders. Geriatr. Gerontol. Int. 16 (1), 17–29. 10.1111/ggi.12724 27018280

[B17] Gordijn S. J. Beune I. M. Thilaganathan B. Papageorghiou A. Baschat A. A. Baker P. N. (2016). Consensus definition of fetal growth restriction: a Delphi procedure. Ultrasound Obstet. Gynecol. 48 (3), 333–339. 10.1002/uog.15884 26909664

[B18] Gordijn S. J. Beune I. M. Ganzevoort W. (2018). Building consensus and standards in fetal growth restriction studies. Best. Pract. Res. Clin. Obstet. Gynaecol. 49, 117–126. 10.1016/j.bpobgyn.2018.02.002 29576470

[B19] Hannan N. J. Jones R. L. White C. A. Salamonsen L. A. (2006). The chemokines, CX3CL1, CCL14, and CCL4, promote human trophoblast migration at the feto-maternal interface. Biol. Reprod. 74 (5), 896–904. 10.1095/biolreprod.105.045518 16452465

[B20] Hao Y. Hao S. Andersen-Nissen E. Mauck W. M. Zheng S. Butler A. (2021). Integrated analysis of multimodal single-cell data. Cell 184 (13), 3573–3587.e3529. 10.1016/j.cell.2021.04.048 34062119 PMC8238499

[B21] Hoo R. Ruiz-Morales E. R. Kelava I. Rawat M. Mazzeo C. I. Tuck E. (2024). Acute response to pathogens in the early human placenta at single-cell resolution. Cell Syst. 15 (5), 30–444.e9. 10.1016/j.cels.2024.04.002 38703772

[B22] Hu C. Li T. Xu Y. Zhang X. Li F. Bai J. (2023). CellMarker 2.0: an updated database of manually curated cell markers in human/mouse and web tools based on scRNA-seq data. Nucleic Acids Res. 51 (D1), D870–d876. 10.1093/nar/gkac947 36300619 PMC9825416

[B23] Ji K. Chen Y. Pan X. Chen L. Wang X. Wen B. (2024). Single-cell and spatial transcriptomics reveal alterations in trophoblasts at invasion sites and disturbed myometrial immune microenvironment in Placenta accreta spectrum disorders. Biomark. Res. 12 (1), 55. 10.1186/s40364-024-00598-6 38831319 PMC11149369

[B24] Jin S. Guerrero-Juarez C. F. Zhang L. Chang I. Ramos R. Kuan C. H. (2021). Inference and analysis of cell-cell communication using CellChat. Nat. Commun. 12 (1), 1088. 10.1038/s41467-021-21246-9 33597522 PMC7889871

[B25] Kaufmann P. Black S. Huppertz B. (2003). Endovascular trophoblast invasion: implications for the pathogenesis of intrauterine growth retardation and preeclampsia. Biol. Reprod. 69 (1), 1–7. 10.1095/biolreprod.102.014977 12620937

[B26] Kim J. S. Romero R. Kim M. R. Kim Y. M. Friel L. Espinoza J. (2008). Involvement of hofbauer cells and maternal T cells in villitis of unknown aetiology. Histopathology 52 (4), 457–464. 10.1111/j.1365-2559.2008.02964.x 18315598 PMC2896045

[B27] Knöfler M. Haider S. Saleh L. Pollheimer J. Gamage T. James J. (2019). Human placenta and trophoblast development: key molecular mechanisms and model systems. Cell Mol. Life Sci. 76 (18), 3479–3496. 10.1007/s00018-019-03104-6 31049600 PMC6697717

[B28] Korsunsky I. Millard N. Fan J. Slowikowski K. Zhang F. Wei K. (2019). Fast, sensitive and accurate integration of single-cell data with harmony. Nat. Methods 16 (12), 1289–1296. 10.1038/s41592-019-0619-0 31740819 PMC6884693

[B29] Li H. Peng H. Hong W. Wei Y. Tian H. Huang X. (2022). Human placental endothelial cell and trophoblast heterogeneity and differentiation revealed by single-cell RNA sequencing. Cells 12 (1), 87. 10.3390/cells12010087 36611882 PMC9818681

[B30] Li Y. Sang Y. Chang Y. Xu C. Lin Y. Zhang Y. (2024). A Galectin-9-Driven CD11c(high) decidual macrophage subset suppresses uterine vascular remodeling in preeclampsia. Circulation 149 (21), 1670–1688. 10.1161/circulationaha.123.064391 38314577

[B31] Liu B. Li F. Wang Y. Gao X. Li Y. Wang Y. (2024). APP-CD74 axis mediates endothelial cell-macrophage communication to promote kidney injury and fibrosis. Front. Pharmacol. 15, 1437113. 10.3389/fphar.2024.1437113 39351084 PMC11439715

[B32] Mercnik M. H. Schliefsteiner C. Fluhr H. Wadsack C. (2022). Placental macrophages present distinct polarization pattern and effector functions depending on clinical onset of preeclampsia. Front. Immunol. 13, 1095879. 10.3389/fimmu.2022.1095879 36713449 PMC9878680

[B33] Nardozza L. M. Caetano A. C. Zamarian A. C. Mazzola J. B. Silva C. P. Marçal V. M. (2017). Fetal growth restriction: current knowledge. Arch. Gynecol. Obstet. 295 (5), 1061–1077. 10.1007/s00404-017-4341-9 28285426

[B34] Okae H. Toh H. Sato T. Hiura H. Takahashi S. Shirane K. (2018). Derivation of human trophoblast stem cells. Cell Stem Cell 22 (1), 50–63.e56. 10.1016/j.stem.2017.11.004 29249463

[B35] Pichot C. S. Arvanitis C. Hartig S. M. Jensen S. A. Bechill J. Marzouk S. (2010). Cdc42-interacting protein 4 promotes breast cancer cell invasion and formation of invadopodia through activation of N-WASp. Cancer Res. 70 (21), 8347–8356. 10.1158/0008-5472.Can-09-4149 20940394 PMC2970640

[B36] Redline R. W. (2015). Classification of placental lesions. Am. J. Obstet. Gynecol. 213 (4), S21–S28. 10.1016/j.ajog.2015.05.056 26428500

[B37] Reyes L. Golos T. G. (2018). Hofbauer cells: their role in healthy and complicated pregnancy. Front. Immunol. 9, 2628. 10.3389/fimmu.2018.02628 30498493 PMC6249321

[B38] Salafia C. M. Minior V. K. Pezzullo J. C. Popek E. J. Rosenkrantz T. S. Vintzileos A. M. (1995). Intrauterine growth restriction in infants of less than thirty-two weeks' gestation: associated placental pathologic features. Am. J. Obstet. Gynecol. 173 (4), 1049–1057. 10.1016/0002-9378(95)91325-4 7485292

[B39] Sferruzzi-Perri A. N. Sandovici I. Constancia M. Fowden A. L. (2017). Placental phenotype and the insulin-like growth factors: resource allocation to fetal growth. J. Physiol. 595 (15), 5057–5093. 10.1113/jp273330 28337745 PMC5538190

[B40] Slyper M. Porter C. B. M. Ashenberg O. Waldman J. Drokhlyansky E. Wakiro I. (2020). Author correction: a single-cell and single-nucleus RNA-seq toolbox for fresh and frozen human tumors. Nat. Med. 26 (8), 1307. 10.1038/s41591-020-0976-3 32587393 PMC7417328

[B41] Stanek J. (2013). Hypoxic patterns of placental injury: a review. Arch. Pathol. Lab. Med. 137 (5), 706–720. 10.5858/arpa.2011-0645-RA 23627456

[B42] Trapnell C. Cacchiarelli D. Grimsby J. Pokharel P. Li S. Morse M. (2014). The dynamics and regulators of cell fate decisions are revealed by pseudotemporal ordering of single cells. Nat. Biotechnol. 32 (4), 381–386. 10.1038/nbt.2859 24658644 PMC4122333

[B43] Tsang J. C. H. Vong J. S. L. Ji L. Poon L. C. Y. Jiang P. Lui K. O. (2017). Integrative single-cell and cell-free plasma RNA transcriptomics elucidates placental cellular dynamics. Proc. Natl. Acad. Sci. U. S. A. 114 (37), E7786–e7795. 10.1073/pnas.1710470114 28830992 PMC5604038

[B44] Turco M. Y. Moffett A. (2019). Development of the human placenta. Development 146 (22), dev163428. 10.1242/dev.163428 31776138

[B45] Vento-Tormo R. Efremova M. Botting R. A. Turco M. Y. Vento-Tormo M. Meyer K. B. (2018). Single-cell reconstruction of the early maternal-fetal interface in humans. Nature 563 (7731), 347–353. 10.1038/s41586-018-0698-6 30429548 PMC7612850

[B46] Wang Y.-H. Lang X.-L. Qing X.-L. (2026). Nanomaterial strategies for mitigating protein misfolding and neuroinflammation in neurodegenerative diseases. Zoological Res. 47 (1), 155–187. 10.24272/j.issn.2095-8137.2025.227 41603025 PMC13276651

[B47] Wedel S. Martic I. Guerrero Navarro L. Ploner C. Pierer G. Jansen-Dürr P. (2023). Depletion of growth differentiation factor 15 (GDF15) leads to mitochondrial dysfunction and premature senescence in human dermal fibroblasts. Aging Cell 22 (1), e13752. 10.1111/acel.13752 36547021 PMC9835581

[B48] WHO Alliance for Maternal and Newborn Health Improvement Late Pregnancy Dating Study Group (2020). Performance of late pregnancy biometry for gestational age dating in low-income and middle-income countries: a prospective, multicountry, population-based cohort study from the WHO alliance for maternal and newborn health improvement (AMANHI) study group. Lancet Glob. Health 8 (4), e545–e554. 10.1016/s2214-109x(20)30034-6 32199122 PMC7091029

[B49] Yang Y. Guo F. Peng Y. Chen R. Zhou W. Wang H. (2021). Transcriptomic profiling of human placenta in gestational diabetes mellitus at the single-cell level. Front. Endocrinol. (Lausanne) 12, 679582. 10.3389/fendo.2021.679582 34025588 PMC8139321

[B50] Zang X. Zhang D. Wang W. Ding Y. Wang Y. Gu S. (2024). Cross-species insights into trophoblast invasion during placentation governed by immune-featured trophoblast cells. Adv. Sci. (Weinh) 11 (42), e2407221. 10.1002/advs.202407221 39234818 PMC11558115

[B51] Zhang T. Bian Q. Chen Y. Wang X. Yu S. Liu S. (2021). Dissecting human trophoblast cell transcriptional heterogeneity in preeclampsia using single-cell RNA sequencing. Mol. Genet. Genomic Med. 9 (8), e1730. 10.1002/mgg3.1730 34212522 PMC8404237

[B52] Zhou W. Wang H. Yang Y. Guo F. Yu B. Su Z. (2022). Trophoblast cell subtypes and dysfunction in the placenta of individuals with preeclampsia revealed by single-cell RNA sequencing. Mol. Cells 45 (5), 317–328. 10.14348/molcells.2021.0211 35289305 PMC9095508

