# Supplementary figures and images for: A FOS/NFKB1-associated Hofbauer cell subset mediates placental niche dysregulation in early-onset fetal growth restriction
Source: Front Cell Dev Biol. 2026 May 29;14:1827600. doi: 10.3389/fcell.2026.1827600 (PMC13260124; doi:10.3389/fcell.2026.1827600)

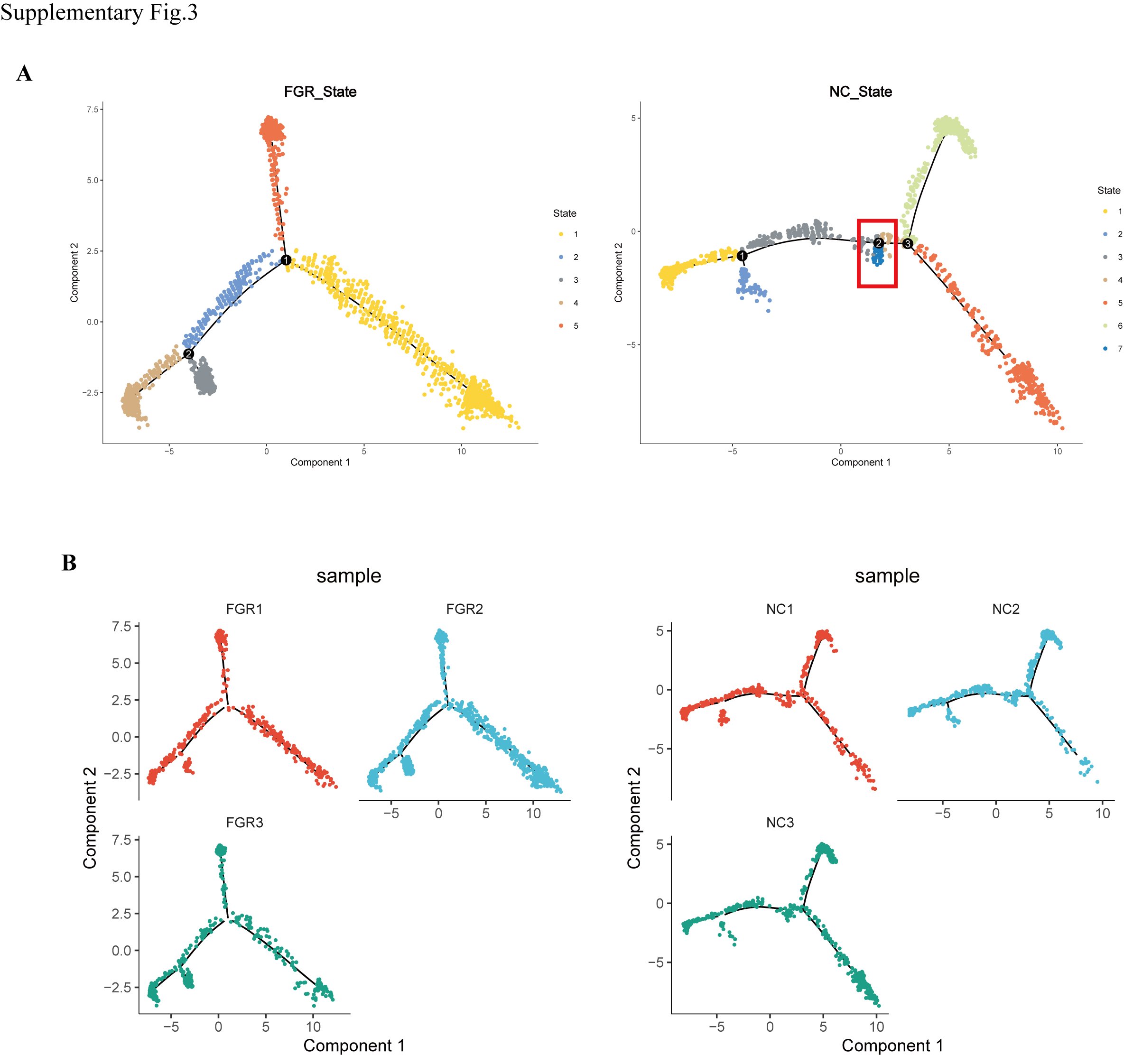

Supplement: Supplementary file 1 [file Image3.tif]

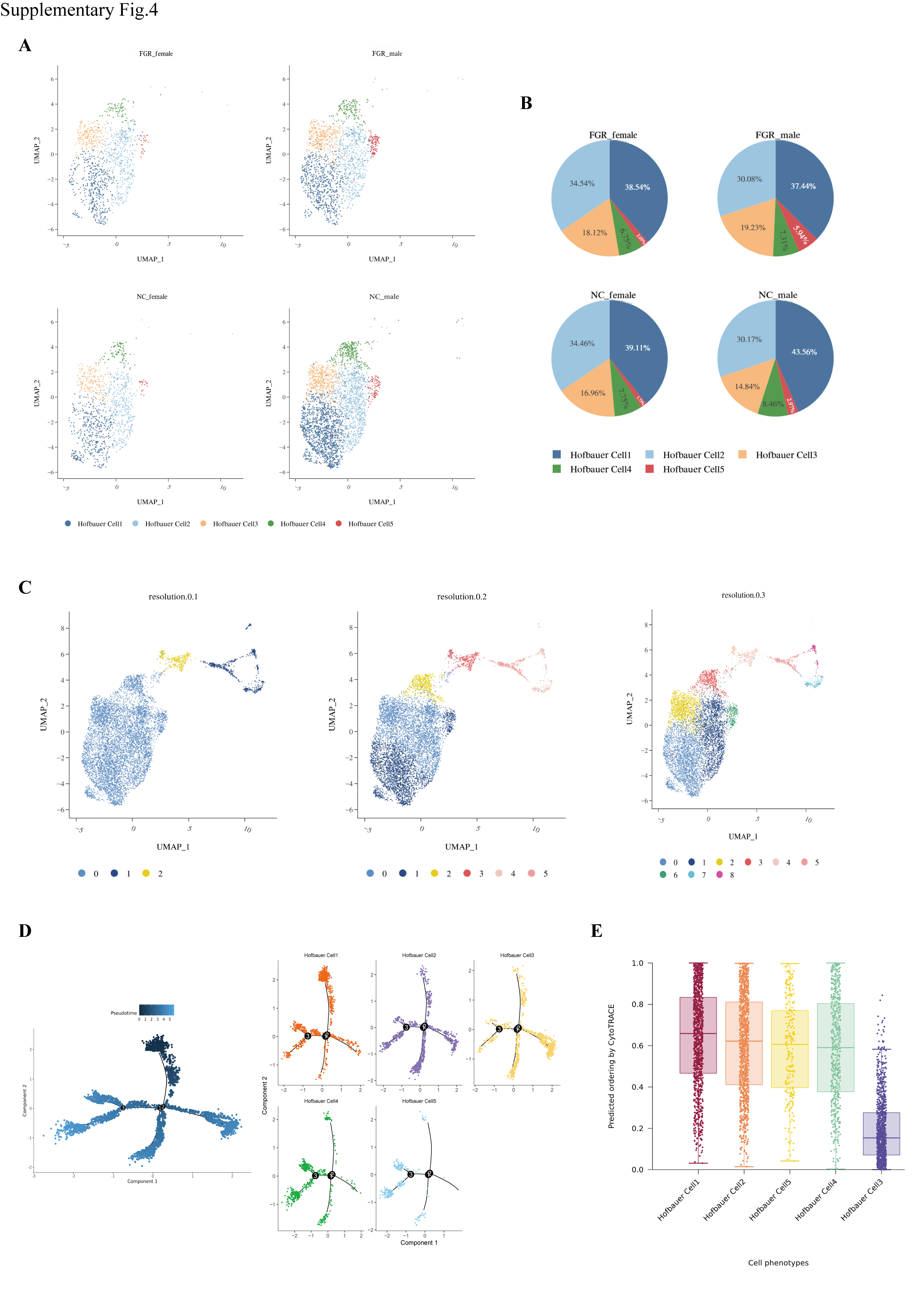

Supplement: Supplementary file 2 [file Image4.tif]

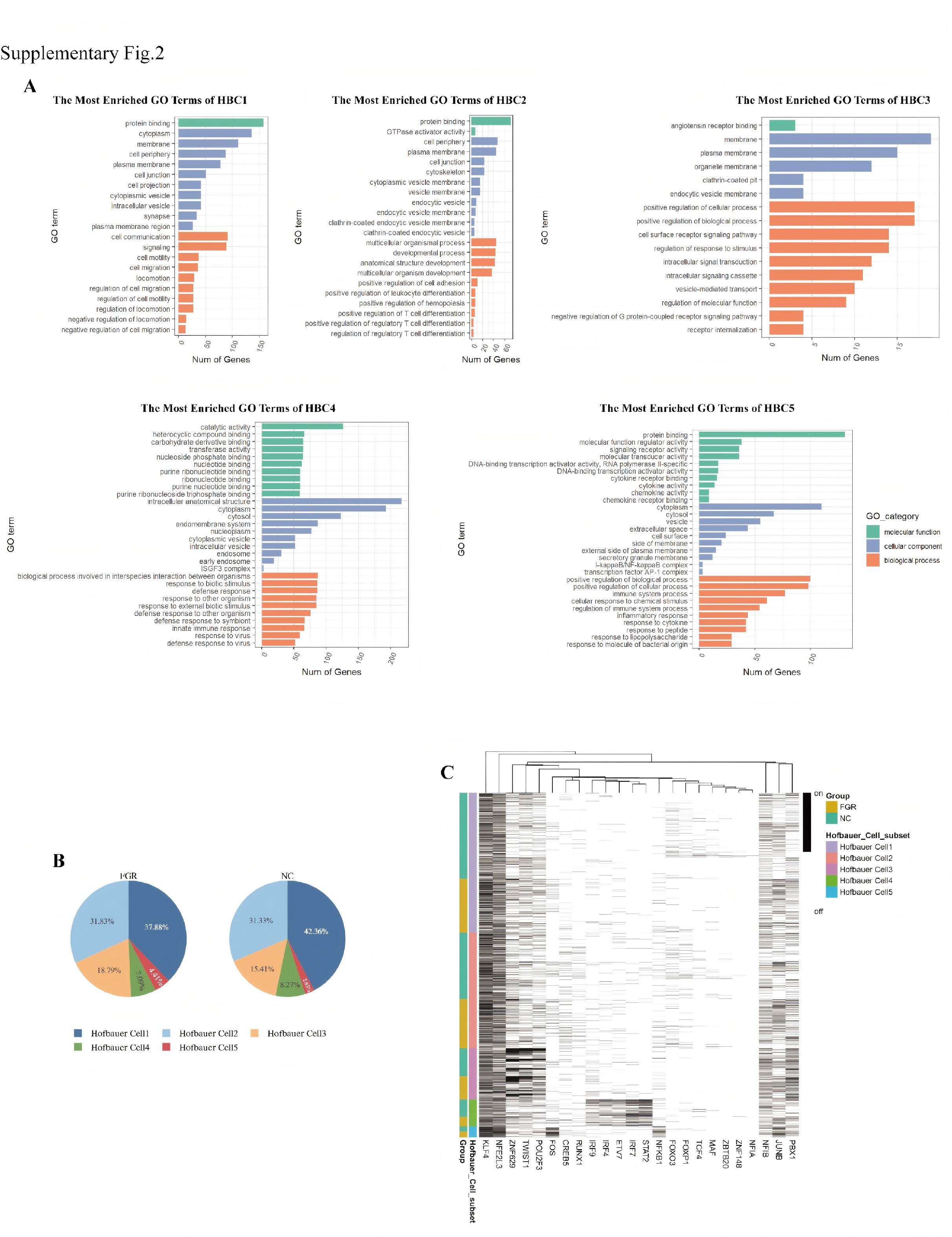

Supplement: Supplementary file 3 [file Image2.jpeg]

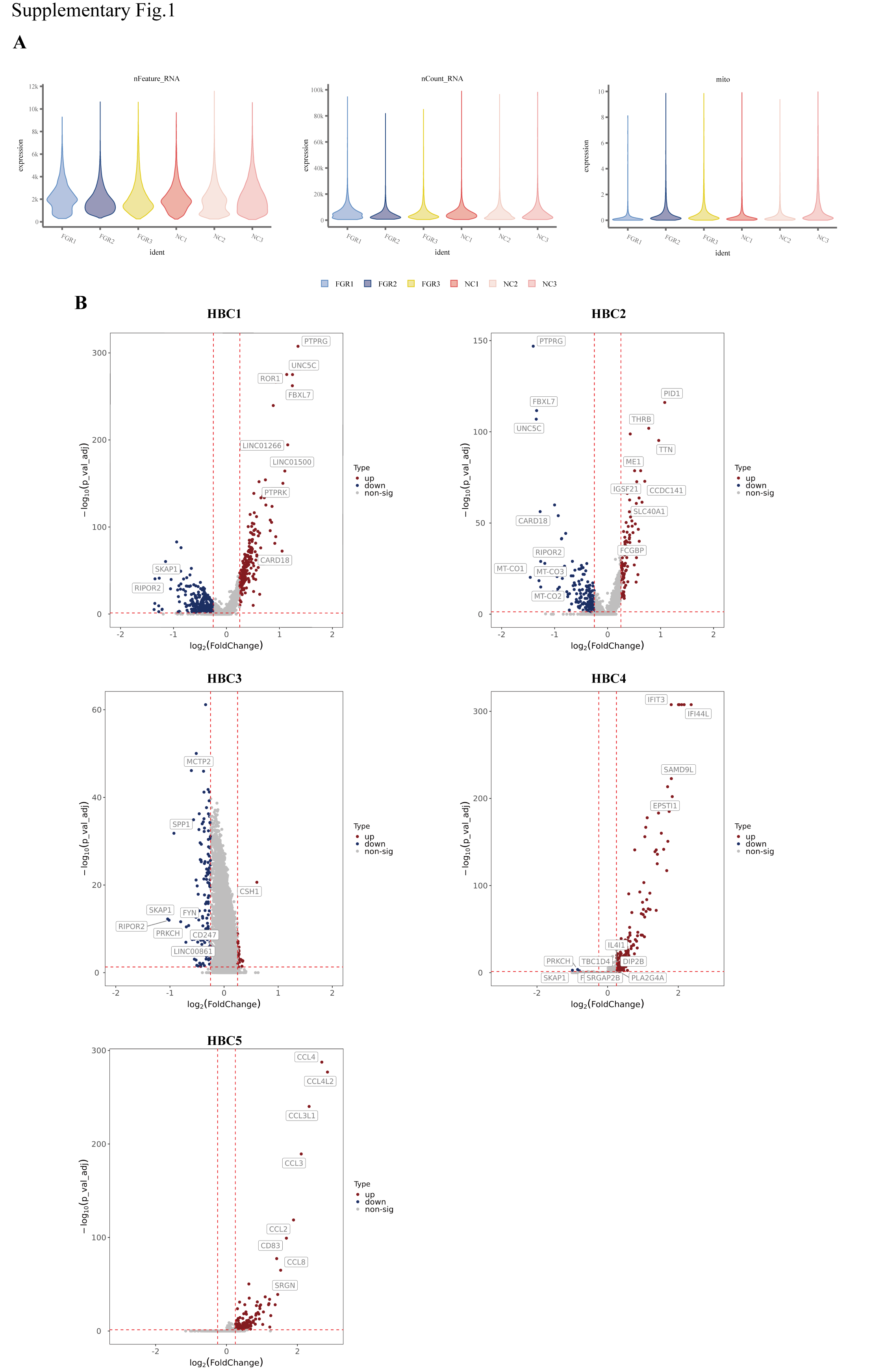

Supplement: Supplementary file 4 [file Image1.tif]
